# Exploring the Emergence of RNA Nucleosides and Nucleotides on the Early Earth

**DOI:** 10.3390/life8040057

**Published:** 2018-11-06

**Authors:** Annabelle Biscans

**Affiliations:** RNA Therapeutics Institute, University of Massachusetts Medical School, Worcester, 01605 MA, USA; annabelle.biscans@umassmed.edu

**Keywords:** origin of life, prebiotic chemistry, nucleotide and nucleoside synthesis

## Abstract

Understanding how life began is one of the most fascinating problems to solve. By approaching this enigma from a chemistry perspective, the goal is to define what series of chemical reactions could lead to the synthesis of nucleotides, amino acids, lipids, and other cellular components from simple feedstocks under prebiotically plausible conditions. It is well established that evolution of life involved RNA which plays central roles in both inheritance and catalysis. In this review, we present historically important and recently published articles aimed at understanding the emergence of RNA nucleosides and nucleotides on the early Earth.

## 1. Introduction

How did life begin? Where did the essential components to life—nucleic acids, proteins, and lipids—come from? To answer these fundamental questions, efforts have been made to understand the prebiotic synthesis of these biomolecules through chemical processes [1]. Nucleic acids, proteins, and lipids share a similar atomic composition, which includes hydrogen, carbon, oxygen, nitrogen, phosphorous, and sulfur. Therefore, we can assume that they have generated from common natural constituents present on Earth. A large number of astrophysicists, physicists, and mathematicians succeeded in identifying the plausible chemical composition of the early Earth using radio telescopes and spacecraft, such as the Atacama large millimeter/submillimeter array (ALMA) implanted in Chile’s Atacama Desert [2] and the Rosetta space probe [3,4]. They reported on morphological, thermal, mechanical, and electrical properties and composition of the surface of satellites, planets, and comets. Apart from water, carbon monoxide, and carbon dioxide, mixtures of fifteen compounds from the chemical groups of alcohols, amines, carbonyls, nitriles, amides, and isocyanates were detected. They showed that the favored geochemical conditions for life to arise involve volcanic activities and/or the impact of meteorites, with complex organic chemistry; several sources of energy; and dynamic light–dark, cold–hot, and wet–dry cycles [5]. Thus, an important amount of chemistry is potentially possible to favor synthesis of biomolecules or their precursors from simple feedstock molecules.

Among the biomolecules, it is well established that RNA may have played a central role in the early evolution of life. Indeed, RNA can not only act as an enzyme and perform catalytic reactions, but it can also store and transfer genetic information [6,7,8,9,10]. Using knowledge on the availability of starting materials on primitive Earth and the geological conditions when life began, the prebiotic synthesis of RNA building blocks has been explored. Miller–Urey experiments [11], which mark the beginning of prebiotic chemistry, inspired Oro et al. to analyze the products formed when ammonium cyanide was refluxed in aqueous solution, leading to the discovery that adenine can be formed by cyanide polymerization [12]. Since this discovery, the prebiotic synthesis of RNA has been intensely investigated. This review gives an overview of the plausible origin of RNA. Different possible routes for the formation of nucleosides and nucleotides, RNA building blocks, under prebiotic conditions will be discussed.

## 2. Prebiotic Synthesis of Nucleotides from the Assembly of a Nucleobase, a Ribose, and a Phosphate

**Figure 1:**
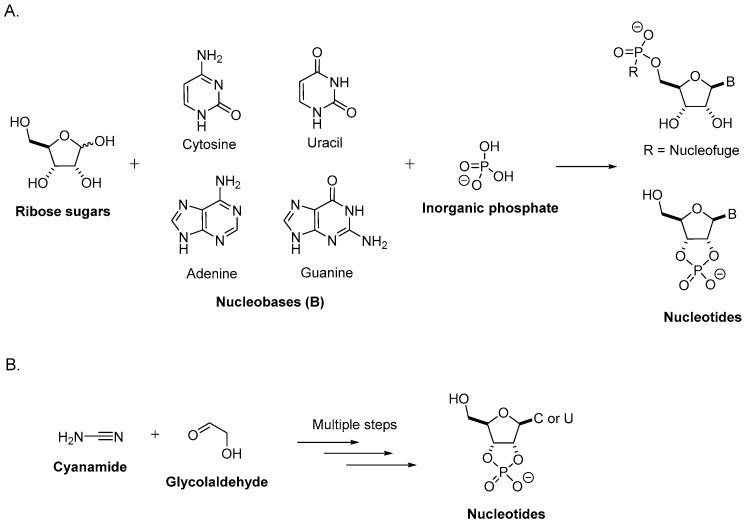
Possible routes for the synthesis of nucleotides. (**A**) The traditional RNA disconnection route, which is based on the hypothesis that nucleotides are formed from a ribose sugar, nucleobases, and inorganic phosphate, each prepared separately and assembled. (**B**) Alternative approach to synthesize nucleotides where sugars and nucleobases are formed during the same process.

First efforts to understand the prebiotic synthesis of ribonucleotides, the building blocks of RNA, have been based on the hypothesis that they should be formed from three distinct entities: a nucleobase (uracil, cytosine, adenine, or guanine), a ribose sugar, and a phosphate, which have been formed separately and combined (Figure 1A) [13].

### 2.1. Sugar Synthesis

Prebiotic chemists suggested that sugar formation relied on the synthesis of formose, discovered by Butlerow in 1861 [14]. This reaction consists of the polymerization of formaldehyde in the presence of calcium hydroxide. For a long time, the mechanism of how this reaction initiates assumed that a homocoupling of formaldehyde occurred to produce glycolaldehyde, later converted in glyceraldehyde. However, such a direct dimerization has been considered chemically unfavored [15]. Recently, Schreiner et al. demonstrated that glycolaldehyde could have been formed from formaldehyde reacting with its isomer hydroxymethylene in the absence of base and solvent at cryogenic temperature [16]. Hydroxymethylene could have been generated from the pyrolysis of glyoxylic acid in the gas phase or on surfaces. During sugar formation, a variety of reactions can occur (myriad aldol; Cannizzaro; or Lobry de Bruyn–Alberda van Ekenstein reactions, which involves transforming an aldose into the ketone isomer or vice versa) leading to a complex mixture of linear and branched aldo- and ketosugars [17]. This uncontrolled reactivity forms ribose with less than 1% yield [18].

**Figure 2:**
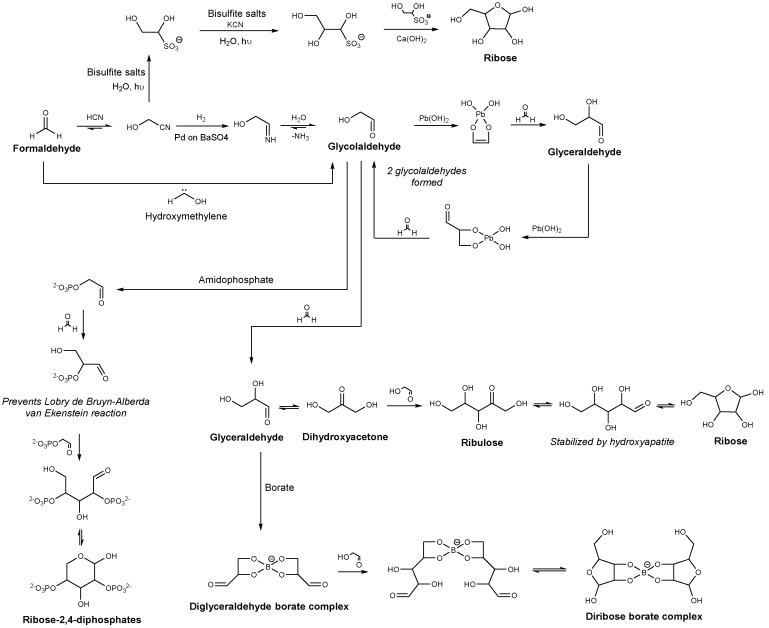
Possible routes favoring ribose formation.

Significant efforts have been made to search efficient plausible prebiotic routes to favor sugar synthesis (Figure 2). Indeed, the formose reaction is a catalyzed reaction [15], and thus many groups focused on the identification of a prebiotic catalyst, which could have explained ribose formation. Zubay et al. showed that more than 30% of the formaldehyde can be converted to a mixture of aldopentoses using a lead catalyzed formose reaction [13,19]. The presence of lead in the incubation mixture also accelerated a number of other reactions including the interconversion of the aldopentoses into ribose. Also, it has been shown that hydroxyapatite, which consists of phosphate and calcium ions, increased ribose formation from formaldehyde and glycolaldehyde in hot water (80 °C) [20]. Hydroxyapatite enhanced cross-aldol reactions and Lobry de Bruyn–Alberda van Ekenstein transformations, utilizing the effective positioning of calcium ions on the surface of hydroxyapatite. 

Moreover, ribose is unstable under the alkaline conditions required for the formose reaction [21]. Borate addition has been investigated as borate could have been available on early Earth and as ribose can form borate complexes, stabilizing the molecule under the harsh prebiotic formation while other sugars would degrade [22,23,24]. However, even though ribose–borate complexes are more stable than other pentoses, the stabilization is modest and an excess of glycolaldehyde over formaldehyde is required to inhibit borate and prevent sugar isomers with no selectivity for ribose. Eschenmoser et al. showed that the variety of products induced by the formose reaction and the destructive effects that the reaction conditions have on the ribose can be significantly suppressed by phosphorylation of glycolaldehyde [25]. Indeed, under alkaline conditions, glycolaldehyde phosphate leads to a simple mixture of tetrose-2,4-diphosphates and hexose-2,4,6-triposphates. The presence of phosphate groups prevents the Lobry de Bruyn–Alberda van Ekenstein reaction and stabilizes the sugars, providing a plausible prebiotic route to the synthesis of ribose if the ribose-2,4-diphospate could later be converted to a 5-phosphate or a 1,5-diphosphate. 

Alternative routes leading to the formation of ribose are possible. Indeed, sugar formation could be coming from hydrogen cyanide irradiated by ultraviolet light in the presence of copper cyanide complexes [26]. Moreover, bisulfite salts could have played a role in sugar formation as certain sulfidic anions could have been available on the early Earth [27]. Sutherland et al. found that bisulfite could be used to form a glycolaldehyde–bisulfite adduct from glycolnitrile. This bisulfite adduct formation, allowing the stabilization of the aldehyde, could have led to the formation of ribose [28,29]. 

In addition, aldoses including ribose pentose sugars have been found in interstellar ice analogs (composed of water, methanol, and ammonia) after their irradiation by ultraviolet light [30]. These results suggest that the formation of numerous sugars, including the ribose, may be possible from photochemical and thermal treatment of cosmic ices in the late stages of the solar nebula. 

Even if some progress has been made to understand the ribose formation under prebiotic conditions, each suggested route presents obstacles, limiting ribose yield and purity necessary to form nucleotides. A selective pathway has yet to be elucidated.

### 2.2. Nucleobase Synthesis

**Figure 3:**
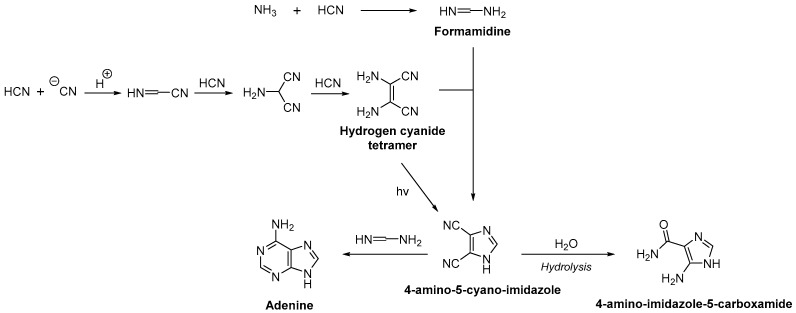
Possible prebiotic synthesis of adenine from hydrogen cyanide.

Starting with the prebiotic purine formation (adenine and guanine), it has been shown that the nucleobase adenine could be formed by mixing hydrogen cyanide and ammonia in solution [31,32]. Indeed, formamidine can be produced by addition of ammonia to hydrogen cyanide. It can then react with hydrogen cyanide tetramer or diaminomaleodinitrile, resulting from hydrogen cyanide polymerization in aqueous solution, to form 4-amino-5-cyano-imidazole. The latter product could then react with a second formamidine molecule to lead to adenine (Figure 3) [33,34,35]. As the hydrolysis of 4-amino-5-cyano-imidazole to form 4-amino-imidazole-5-carboxamide can occur, these results suggest that a high concentration of hydrogen cyanide and ammonia should have been present on Earth. Even though hydrogen cyanide could be found in high concentration in frozen environments [31,36], a significant amount of ammonia on Earth is questionable. Ferris and Orgel suggest an alternative route where the production of 4-amino-5-cyano-imidazole could have been possible by photochemical isomerization of hydrogen cyanide tetramer, a mechanism that does not involve ammonia [32].

Other hypotheses on adenine and purine accumulation on Earth have been discussed. Miyakama et al. suggest that purines have been formed in the atmosphere in the absence of hydrogen cyanide [37]. They reported that guanine could have been generated from a gas mixture (nitrogen, carbon monoxide, and water) after cometary impacts. Also, it has been proposed that adenine was formed in the solar system (outside of Earth) and brought to Earth by meteorites, given the fact that adenine was found in significant quantity in carbonaceous chondrites [38].

Most of the work on the prebiotic synthesis of pyrimidines (cytosine and uracil) suggest reactions between cyanoacetylene or its hydrolysis product, cyanoacetaldehyde, and cyanate ions, cyanogen, or urea [39,40,41,42]. Indeed, in concentrated urea solution, which could have been found in an evaporating lagoon on the early Earth, cyanoacetaldehyde could have reacted to form cytosine with a yield of 30–50%, from which uracil could be generated by hydrolysis [41]. As the cyanoacetylene can be formed when an electrical discharge is passed through a mixture of nitrogen and methane [43], and as its hydrolysis into cyanoacetaldehyde occurs spontaneously [39], these molecules can be considered prebiotic [44]. For example, pyrimidine formation has been observed when an energy source was applied on a urea solution in the presence of methane and nitrogen at low temperatures [45].

### 2.3. Nucleoside Synthesis from Sugars and Nucleobases

The synthesis of nucleosides from sugars and nucleobases in prebiotic conditions is one of the major difficulties encountered, when attempting to resolve the early formation of nucleosides. The reaction between the ribose and nucleobase is thermodynamically unfavorable, leading to poor yields and little selectivity [46]. Only a few examples showing successful synthesis have been reported in the literature. 

The formation of adenosine (4% yield) has been observed from the condensation of adenine with ribose in the presence of inorganic salts, providing complex mixtures of purine ribosides [47]. However, regioselectivity problems were encountered owing to the reactivity of all N atoms of the purine skeleton. To overcome this regiospecificity limitation, Becker et al. showed that *N*-formamidopyrimidines could be used to generate purine nucleosides with absolute nucleobase regioselectivity [48]. Recently, they reported a plausible prebiotic synthesis of formamidopyrimidines, which can be generated from 5-nitroso-pyrimidine in the presence of formic acid and elementary metals (Ni or Fe). When combined with ribose, formamidopyrimidines can react and lead to efficient production of canonical and non-canonical purine bases in parallel [49].

In addition, adenine nucleoside phosphate has been formed from the direct coupling reaction of cyclic carbohydrate phosphate with the free nucleobase. The reaction is stereoselective and regioselective, giving the N-9 nucleotide as a major product [50]. It is unknown if pyrimidine nucleosides that could be formed with the same strategy as pyrimidines are more resistant to ribosylation [46]. Moreover, a feasible prebiotic pathway to synthesize both purine and pyrimidine simultaneously under the same conditions in an aqueous microdroplet containing ribose, phosphoric acid, nucleobases, and small amounts of magnesium ion has been reported [51]. Indeed, microdroplets allow organization of the molecules at the air–water interface of their surfaces, which possess a strong electrical field [52], diminishing the thermodynamic barrier for chemical reactions [53].

Even though important efforts are made to determine the possible prebiotic conditions for the nucleoside formation from sugars and nucleobases, this strategy leads to significant problems and an alternative approach has been suggested to form nucleosides [54].

## 3. The Revisited Approach for the Synthesis of Nucleosides

The alternative approach for the synthesis of nucleosides is based on the hypothesis that nucleobases and sugars emerged from a common precursor. This would mean that both the sugar and the nucleobase are formed during the same process (Figure 1B). 

### 3.1. Pyrimidine Nucleoside Synthesis

Many years ago, Orgel et al. reported the synthesis of α-cytidine from ribose, cyanamide, and cyanoacetylene in aqueous solution. They showed that the replacement of the ribose by a ribose-5-phosphate allows the obtention of α-cytidine-5′-phosphate, which can be photoanomerized into β-cytidine-5′-phosphate required for RNA synthesis [55]. However, during this process, the yields were low (5%) and too many side products were formed to consider this route as prebiotic. Moreover, the photoanomerization destroyed most of the nucleosides [56,57]. Therefore, the formation of pyrimidines under plausible prebiotic conditions had to be further investigated. 

Remarkably, one nucleotide, β-cytidine-2′-3′-cyclic phosphate, showed great stability under irradiation; only partial conversion to the corresponding uridine was observed [58]. Irradiation could thus provide a mechanism to destroy undesired products and partially convert cytidine into uridine. Therefore, the synthesis of this nucleotide raised a lot of interest. However, the determination of the pathway to obtain this cyclic phosphate under prebiotic conditions was a challenge. 

**Figure 4:**
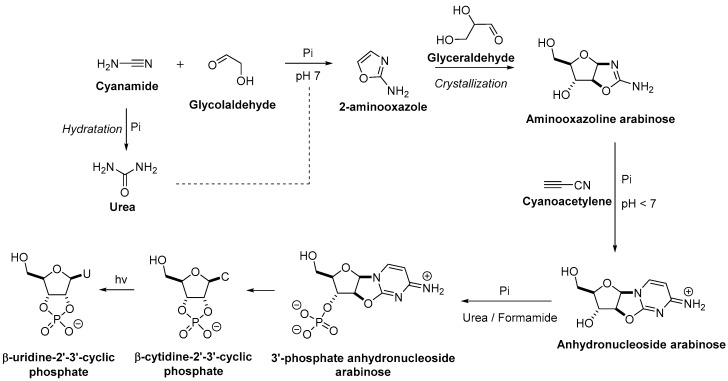
Possible prebiotic route for the synthesis of pyrimidine nucleotides. U = uridine; C = cytidine; Pi = inorganic phosphate.

Inspired by previously reported work of Navigary et al., who demonstrated that β-cytidine-2′-3′-cyclic phosphate can be obtained from the 3′-phosphate of the anhydronucleoside arabinose (which was prepared by conventional synthesis) [59], Sutherland et al. showed that it is possible to prebiotically obtain the desired 3′-phosphate of the anhydronucleoside arabinose from the corresponding arabinose in the presence of urea melts and formamide (Figure 4) [58]. 

The latter intermediate can be formed from the aminooxazoline arabinose and cyanoacetylene [55]. The presence of a phosphate during the reaction is essential to induce the right reactivity and to allow pH buffering, making the conversion of the aminooxazoline arabinose into the anhydronucleoside arabinose clean and with good yield (>90%).

Aminooxazoline arabinose could be formed by the reaction of 2-aminooxazole and glyceraldehyde in excellent yields [60]. This reaction supports construction of a five-carbon pentose backbone with complete furanosyl selectivity and regiospecific glycosylation in one step. The differential solubilities of pentose aminooxazolines facilitated a direct crystallization of pure compounds from the reaction mixture [60,61]. 

Synthesis of 2-aminooxazole by reaction of glycolaldehyde with cyanamide was achieved [62]. Once again, the presence of a phosphate during this step was crucial to obtain good yields (~90%). The phosphate mediated a neutral pH and first catalyzed the production of 2-aminooxazole. It also permitted the hydration of the excess cyanamide to urea, which in turn catalyzed the formation of 2-aminooxazole [63]. This synthetic route can be truly considered as prebiotic as the pyrimidines were formed from glycolaldehyde and cyanamide.

**Figure 5:**
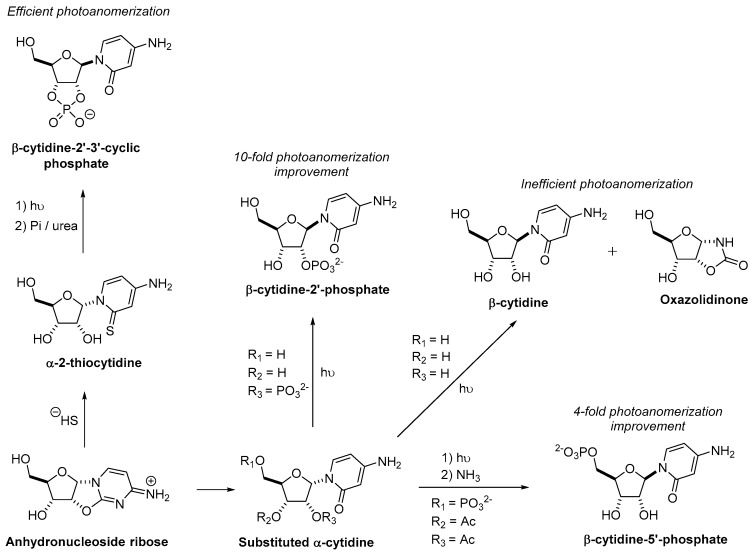
Alternatives to improve photoanomerization α→β for the formation of pyrimidine nucleotides. Pi = inorganic phosphate; Ac = acetyl.

Another route to obtain β-cytidine-2′-3′-cyclic phosphate by photoanomerization from ribose aminooxazoline instead of arabinose aminooxazoline was investigated (Figure 5). In fact, ribose aminooxazoline has a greater propensity to crystallize than its stereoisomers, making it extremely attractive in prebiotic chemistry. As mentioned previously, Orgel et al. reported that the photoanomerization of α-cytidine into β-cytidine results in a low yield (about 5%) [55]. Indeed, the low yield can be partially explained by a combination of nucleobase loss and oxazolidinone formation [56]. To improve this process, it has been suggested to incorporate a 2′-phosphate, leading to a 10-fold improvement of the photoanomerization [64]. However, a prebiotic synthesis of α-cytidine-2′-phosphate has not yet been demonstrated. Moreover, acetylation of α-cytidine-5’-phosphate was investigated to block oxazolidinone formation and a four-fold improvement of photoanomerization to produce β-cytidine-5′-phosphate has been observed [65]. Another solution to the inefficient photoisomerization has been found, and consisted of reacting anhydronucleoside ribose with hydrosulfide to form α-2-thiocytidine. Irradiation of the latter compounds led to the β stereochemistry. In addition, phosphorylation in the presence of urea produced the 2′-3′-cyclic phosphate nucleotide and converted the nucleobase thiocarbonyl to a carbonyl in one step to form β-cytidine-2′-3′-cyclic phosphate [66].

These two routes, exploring both ribose and arabinose variants, are key working examples for understanding pyrimidine formation under prebiotic conditions.

### 3.2. Purine Nucleoside Synthesis

**Figure 6:**
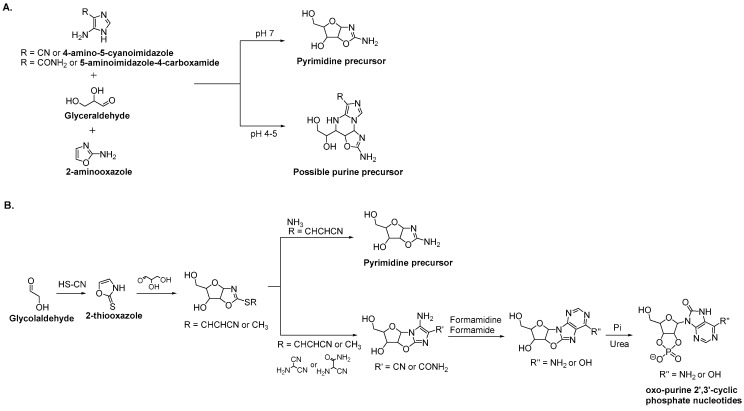
Possible prebiotic formation of both pyrimidine and purine precursors from a common environment. (**A**) Possible purine precursor from 4-amino-5-cyanoimidazole or 5-aminoimidazole-4-carboxamide, 2-aminooxazole, and glyceraldehyde. (**B**) Possible pyrimidine and oxo-purine from 2-thiooxazole. Pi = inorganic phosphate.

Possible routes for purine formation still have to be investigated. However, preliminary studies provide potential leads. Indeed, a purine precursor has been successfully assembled from 4-amino-5-cyanoimidazole or 5-aminoimidazole-4-carboxamide, 2-aminooxazole, and glyceraldehyde (Figure 6A). This Mannich-type reactivity results in N^9^-glycolysation with absolute regiospecificity [67]. This route is particularly interesting because it provides the opportunity to produce both purine and pyrimidine precursors from the same environment. Moreover, the preference between purine or pyrimidine precursor formation can be controlled by pH. At pH 7, pentoses aminooxazolines (pyrimidine precursors) are predominant, whereas at pH 4–5, purine precursors are dominant. At a pH between 5 and 7, a mixture of both precursors are observed. 

Another prebiotically plausible reaction for the synthesis of both pyrimidine and purine nucleotides from an oxazoline scaffold has been reported (Figure 6B). An oxazolidinone thione provided the chemical differentiation required for divergent pyrimidine and 8-oxo-purine nucleotide synthesis from one common precursor, the 2-thiooxazole [68]. Even though the transformation of the oxo-purine 2′,3′-cyclic phosphate nucleotides to the canonical nucleotides remains to be determined, it is possible that oxo-purine nucleotides were tolerated during template-directed RNA synthesis [69].

While promising, these proposed synthetic pathways demand further investigation.

## 4. Conclusions and Outlook

The role of RNA in the origin of life is well established, and understanding how RNA emerged on the early Earth is one of the first steps in understanding the origins of life. Despite great efforts and impressive advancements in the study of nucleoside and nucleotide abiogenesis, further investigation is necessary to explain the gaps in our understanding of the origin of RNA.

The comprehension of nucleotide formation under prebiotic conditions is only one of the steps to understand the complex production of RNA as nucleotides must be oligomerized to generate RNA. Assuming that RNA must be 5′-3′-linked, regioselectivity issues have to be overcome. Polymerization of activated nucleotides has been studied intensely as a model for non-enzymatic oligomerization of RNA and has been considered a plausible scenario for the emergence of RNA during the origin of life [70,71,72,73,74,75]. Furthermore, for its genetic role to be realized, RNA must be able to evolve and replicate [6,76]. Unfortunately, the chemical processes that sustain RNA oligomerization and replication remain unclear [77].

Other than RNA, cells require various chemical subsystems, including peptides for functional support and lipids for compartmentalization. The assumption that one subsystem came first and then generated the others is debated [78,79,80]. Consequently, a search for a chemistry that can concurrently deliver nucleotides, peptides, and lipids or for chemistries that can be compatible with each other within the same geochemical environment could provide the most compelling explanation for the origins of life.
